# Reflexed cryptococcal antigenaemia detection rates in the Western Cape, South Africa

**DOI:** 10.4102/sajhivmed.v26i1.1676

**Published:** 2025-04-23

**Authors:** Naseem Cassim, Manuel P. da Silva, Lindi-Marie Coetzee

**Affiliations:** 1 Wits Diagnostic Innovation Hub, Faculty of Health Sciences, University of the Witwatersrand, Johannesburg, South Africa; 2 National Priority Programme, National Health Laboratory Service, Johannesburg, South Africa

**Keywords:** HIV, cryptococcal disease, reflexed, cryptococcal antigenaemia, detection rate

## Abstract

**Background:**

Reflexed cryptococcal antigenaemia (CrAg) testing has been offered on remnant CD4 specimens with a count < 100 cells/µL from 2017 in South Africa. The Western Cape is the only province to introduce CrAg testing for counts of 100 cells/µL to 200 cells/µL.

**Objectives:**

The objective of this study was to assess the reflexed CrAg detection rate in the Western Cape.

**Method:**

The retrospective analysis of laboratory data for reflexed CrAg testing was conducted between September 2022 and May 2024. The CrAg detection rate was reported for the following CD4 categories at the provincial, district, and sub-district levels: (1) < 100 cells/µL, (2) ≥ 100 cells/µL – ≤ 200 cells/µL, and (3) ≤ 200 cells/µL.

**Results:**

Data are reported for 80 809 specimens, with a CrAg detection rate of 4.0% for a CD4 ≤ 200 cells/µL compared to 6.2% for a count < 100 cells/µL. For a count of ≥ 100 cells/µL – ≤ 200 cells/µL, a CrAg detection rate of 2.1% was reported. The district CrAg detection rate for counts ≤ 200 cells/µL ranged from 1.4% (Central Karoo) to 5.9% (Cape Winelands). Excluding subdistricts without CrAg-positive specimens, the detection rate ranged from 1.3% (Beaufort West) to 8.3% (Swartland).

**Conclusion:**

The study findings of a CrAg detection rate of 4.0% in the Western Cape province justifies their decision to extend reflexed screening to a threshold of 200 cells/µL. However, most CrAg-positive specimens were identified for a count < 100 cells/µL. Intensified approaches to find CrAg-positive patients with a count ≤ 200 cells/µL are required.

**What this study adds:** This study provides insights into reflexed cryptococcal antigenaemia (CrAg) detection rates in the Western Cape province, where testing was extended to counts ≤ 200 cells/µL. The CrAg detection was higher with a count < 100 cells/µL, but the threshold of < 200 cells/uL should be considered if resources are available.

## Introduction

The National Health Laboratory Service (NHLS) is the largest pathology service in South Africa, responsible for providing diagnostic services to the national and provincial health departments.^1^ This is achieved through a network of laboratories that provide access to over 80% of the population.^1^

There are 7.7 million people living with HIV (PLHIV) in South Africa, of whom 77% were on antiretroviral therapy (ART) in 2023.^2^ Despite the scale-up of ART, local reports indicate that the proportion of patients presenting with advanced HIV disease (AHD), defined as a CD4 count ≤ 200 cells/µL or the clinical manifestation of stage III/IV HIV disease, remains consistently high, representing a large and avoidable burden of morbidity.^3,4,5^ Ford et al. reported that a substantial proportion of patients are still at risk of death because of progressing to AHD, which has remained constant despite ongoing improvements in access to ART.^6^ A local study reported that between 2005 to 2011, the proportion of patients entering care with AHD declined from 46.8% to 35.6%.^3^ In comparison, between 2011 and 2016, the proportion of patients entering ART with AHD has remained relatively unchanged.^3^ A Western Cape study analysed CD4 distributions in adults 16 years and older from a derived province-wide HIV cohort between 2008 and 2017.^7^ By 2017, 22.8% of patients reported a CD4 count < 200 cells/µL.^7^

The World Health Organization (WHO) recommended a package of interventions for PLHIV presenting with AHD that include screening, treatment and/or prophylaxis for major opportunistic infections, rapid ART initiation, and intensified adherence support interventions.^8^ The leading causes of mortality among adults with AHD globally include tuberculosis (TB), severe bacterial infections, cryptococcal meningitis (CM), toxoplasmosis and *Pneumocystis jirovecii* pneumonia.^8^

In South Africa, the national reflexed cryptococcal antigenaemia (CrAg) testing programme has been in place since 2017 for PLHIV with a CD4 count < 100 cells/µL.^9,10,11^ The current local HIV guidelines recommend that a reflexed CrAg test should be done automatically for all CD4 counts < 100 cells/µL.^4^ Patients with a CrAg-positive test in the absence of symptoms of meningitis and a negative lumbar puncture for CM can be initiated on ART.^4^ Those with confirmed CM should defer ART for 4 to 6 weeks until antifungal treatment has been completed.^4^ For patients that are not diagnosed with reflexed screening, the healthcare worker can still request a CrAg test for patients with a clinical suspicion of meningitis, which includes symptoms such as headache, confusion, fever, neck stiffness or visual disturbances (provider-initiated).^4,10,11^

The Southern African HIV Clinicians Society (SAHCS) guidelines recommended that reflexed CrAg testing should be extended to include specimens with a CD4 count between 100 cells/µL and 200 cells/µL.^9^ Several studies reported that CrAg screening for PLHIV with a CD4 < 100 cells/µL would miss cases of CM in patients with a count between 100 cells/µL and 200 cells/µL.^6,9,12^ Furthermore, it was reported that extending reflexed CrAg screening to a CD4 threshold of 200 cells/µL would provide an important mortality benefit.^9^ The 2022 WHO guidelines recommended CrAg screening for a CD4 cell count < 100 cells/µL,^13^ and offer a recommendation that CrAg screening should be considered at a higher CD4 threshold of 200 cells/µL.^13^ Local guidelines recommend an induction regimen for CM which consists of 1 week of amphotericin B deoxycholate and flucytosine followed by 1 week of fluconazole.^9^ Similarly, public sector guidelines indicate that patients with a lumbar puncture cerebrospinal fluid (CSF) CrAg-positive should receive amphotericin B and fluconazole.^4,14,15^

Cost-effectiveness studies conducted in Cambodia, South Africa, Uganda, and Vietnam for reflexed CrAg testing with a count of < 100 cells/µL have reported that this approach is extremely cost-effective, even using a low-range prevalence estimate of 2%.^16,17,18,19,20^ However, Meya et al. reported that above a prevalence of approximately 3%, the cost of amphotericin deoxycholate treatment of people unmasking ART-associated CM ($245 per person) is greater than the costs of screening and treating with pre-emptive fluconazole (excluding hospitalisation costs).^19^ In contrast, a Botswana study reported that CrAg screening for individuals with CD4 101–200 cells/µL was estimated to have a modest impact, involve additional costs and be less cost-effective than screening populations with CD4 counts ≤ 100 cells/µL.^21^ There appears to be limited data showing the cost-effectiveness and detection rates of extending CrAg screening to a CD4 threshold of 200 cells/µL in a sub-Saharan African context.^22^

The Western Cape province implemented the amended criteria for reflexed testing for CrAg at all CD4 laboratories from 15 August 2022.^9,13,23^ The circular recommended that all CD4 specimens from public health facilities from patients of all ages should receive a reflexed CrAg for a CD4 ≤ 200 cells/µL.^23^ The lack of prevalence data to support extending CrAg testing to a CD4 threshold of 200 cells/µL in a country such as South Africa, that continues to have a high HIV burden, is a challenge.^2^ The availability of specimen-level data for the Western Cape province provides an opportunity to assess the CrAg detection rates at various CD4 categories.

### Objectives

The objectives of this study were to assess the reflexed CrAg detection rate in the Western Cape province in South Africa, where testing was extended to a CD4 threshold of 200 cells/µL at the provincial, district and sub-district levels.

## Research methods and design

### Context

Data were reported for reflexed CrAg testing performed in the Western Cape province by CD4 laboratories within the NHLS.^1^ These laboratories use a national laboratory information system (LIS) rule-based algorithm to identify specimens that require reflexed CrAg testing.

### Study design

The retrospective analysis of laboratory data for reflexed CrAg testing was conducted between September 2022 and May 2024 for a CD4 threshold of 200 cells/µL.

### Data preparation

The data extract was provided by the NHLS laboratory data repository and included the following variables: (1) episode number; (2) result authorisation date; (3) age (in years); (4) gender; (5) province; (6) health district; (7) sub-district; (8) health facility; (9) testing laboratory; (10) absolute CD4 count; and (11) CrAg result. The year and month were extracted from the result authorisation date. The absolute CD4 count was categorised as follows: (1) < 100 cells/µL; (2) ≥ 100 cells/µL – ≤ 125 cells/µL; (3) > 125 cells/µL – ≤ 150 cells/µL; (4) > 150 cells/µL – ≤ 175 cells/µL; and (5) > 175 cells/µL – ≤ 200 cells/µL. In addition, the CD4 count was also categorised as < 100 cells/µL, ≥ 100 cells/µL – ≤ 200 cells/µL, and ≤ 200 cells/µL. The CrAg result was reported as either ‘Negative’ or ‘Positive’. The data sets were prepared and analysed using SAS 9.4 (SAS Institute, Cary, North Carolina, United States). Choropleth maps were created using ArcGIS (Environmental Systems Research Institute [ESRI], Redlands, California, United States). Shapefiles were obtained from the Municipal Demarcation Board.^24^

### Statistical analysis

The overall CrAg detection rate was determined by CD4 category, with specimen volumes and the number of positive CrAg specimens indicated. The 95% confidence interval (CI) for the CrAg detection rate was also reported. The overall CrAg detection rate was assessed at the health district and sub-district levels. The list of sub-districts is described in Online Appendix 1. The sub-district CrAg detection rates were reported as choropleth maps with data categorised into the following bins: (1) 0% – 2.9%; (2) 3.0% – 5.7%; (3) 5.8% – 8.7%; and (4) 8.8% – 11.4%. The bins were assigned a colour ramp from green (lowest detection rate) to red (highest detection rate).

### Ethical considerations

Ethical clearance to conduct this study was obtained from the University of Witwatersrand, Human Research Ethics Committee (HREC)(Medical) (reference no.: M220163). Anonymised secondary laboratory data were used. Patient consent was not required.

## Results

Data are reported for 80 809 specimens that received reflexed CrAg testing.

### Overall cryptococcal antigen detection rate

Overall, there were 38 388 (47.5%) specimens with a count of < 100 cells/µL and 42 419 (52.5%) with a count of ≥ 100 cells/µL – ≤ 200 cells/µL (Table 1). A CD4 count of ≥ 100 cells/µL – ≤ 125 cells/µL was reported for 13.1% of the specimens, > 125 cells/µL – ≤ 150 cells/µL for 13.0%, > 150 cells/µL – ≤ 175 cells/µL for 13.4%, and > 175 cells/µL – ≤ 200 cells/µL for 13.0% of the specimens. Most specimens with a positive CrAg result were for a count < 100 cells/µL (72.7%). For a CD4 count of ≥ 100 cells/µL – ≤ 200 cells/µL, a CrAg detection rate of 2.1% (95%CI: 2.0–2.2) was reported. An overall CrAg detection rate of 4.0% (95%CI: 3.9–4.2) was reported for an overall CD4 count ≤ 200 cells/µL. This increased to 6.2% (95%CI: 5.9–6.4) for a CD4 < 100 cells/µL. A CrAg detection rate of 3.0% was reported for a count of ≥ 100 cells/µL – ≤ 125 cells/µL, 2.2% for > 125 cells/µL – ≤ 150 cells/µL, 1.8% for > 150 cells/µL – ≤ 175 cells/µL, and 1.4% for > 175 cells/µL – ≤ 200 cells/µL.

**TABLE 1:** Reflexed cryptococcal antigenaemia (CrAg) detection rate for various CD4 categories for a count of ≤ 200 cells/µL in the Western Cape between September 2022 and May 2024.

CD4 category (cells/µL)	Specimens		CrAg-positive specimens	CrAg detection rate	
	*n*	%	*n*	%	95%CI
< 100	38 388	47.5	2368	6.2	5.9–6.4
≥ 100 – ≤ 125	10 575	13.1	316	3.0	2.7–3.3
> 125 – ≤ 150	10 519	13.0	232	2.2	1.9–2.5
> 150 – ≤ 175	10 856	13.4	191	1.8	1.5–2.0
> 175 – ≤ 200	10 469	13.0	149	1.4	1.2–1.7
≥ 100 – ≤ 200	42 419	52.5	888	2.1	2.0–2.2
Overall	80 807	100.0	3256	4.0	3.9–4.2

Note: The 95% confidence interval (CI) for the CrAg detection rate is indicated.

CrAg, cryptococcal antigenaemia; CI, confidence interval.

### Health district cryptococcal antigen detection rate

For a count < 100 cells/µL, the CrAg detection rate ranged from 2.5% (95%CI: 1.3–4.4) in the Central Karoo to 9.2% (95%CI: 8.4–10.0) for the Cape Winelands. Three districts reported a CrAg detection rate above the provincial rate for a count < 100 cells/µL, namely Cape Winelands (9.2%), West Coast (8.3%) and Garden Route (6.4%). The CrAg detection rate ranged from 0.4% (95%CI: 0.0–1.5) for the Central Karoo to 2.9% (95%CI: 2.5–3.4) for the Cape Winelands for a CD4 of ≥ 100 cells/µL – ≤ 200 cells/µL (Table 2). Only two districts reported a CrAg detection rate for a count of ≥ 100 cells/µL – ≤ 200 cells/µL above the provincial value (Cape Winelands and West Coast). For a count < 100 cells/µL, only the Central Karoo district reported a CrAg detection rate below 3%. In comparison, for a count of ≥ 100 cells/µL – ≤ 200 cells/µL, no districts exceeded 3%. For a count ≤ 200 cells/µL, the CrAg detection rate ranged from 1.4% (95%CI: 0.7–2.4) for the Central Karoo to 5.9% (95%CI: 5.5–6.4) in the Cape Winelands. All except the Central Karoo reported a CrAg detection rate of ≥ 3% for a count of ≤ 200 cells/µL.

**TABLE 2:** Reflexed cryptococcal antigenaemia (CrAg) detection rate at the health district level for various CD4 categories for a count < 100 cells/µL, ≥ 100 cells/µL – ≤ 200 cells/µL, and ≤ 200 cells/µL in the Western Cape between September 2022 and May 2024.

Health district	< 100 cells/µL					≥ 100 cells/µL – ≤ 200 cells/µL					≤ 200 cells/µL				
	Specimens		CrAg+	CrAg detection rate		Specimens		CrAg+	CrAg detection rate		Specimens		CrAg+	CrAg detection rate	
					
	*n*	%	*n*	%	95%CI	*n*	%	*n*	%	95%CI	*n*	%	*n*	%	95%CI
Cape Winelands	5015	13.1	460	**9.2**	8.4–10.0	5576	13.1	164	2.9	2.5–3.4	10 591	13.1	624	**5.9**	**5.5–6.4**
Central Karoo	438	1.1	11	**2.5**	1.3–4.4	494	1.2	2	0.4	0.0–1.5	932	1.2	13	**1.4**	**0.7–2.4**
City of Cape Town	24 551	64.0	1323	**5.4**	5.1–5.7	27 327	64.4	532	1.9	1.8–2.1	51 878	64.2	1855	**3.6**	**3.4–3.7**
Garden Route	4156	10.8	266	**6.4**	5.7–7.2	4601	10.8	87	1.9	1.5–2.3	8757	10.8	353	**4.0**	**3.5–4.5**
Overberg	1896	4.9	114	**6.0**	5.0–7.2	2088	4.9	37	1.8	1.3–2.4	3984	4.9	151	**3.8**	**3.2–4.4**
West Coast	2332	6.1	194	**8.3**	7.2–9.5	2333	5.5	66	2.8	2.2–3.6	4665	5.6	260	**5.6**	**4.6–6.3**
Overall	38 388	1000	2368	**6.2**	5.9–6.4	42 419	100.0	888	2.1	2.0–2.2	80 807	100.0	3256	**4.0**	**3.9–4.2**

Note: Bold formatting was used to identify CrAg detection rates ≥ 3%.

CrAg, cryptococcal antigenaemia; CI, confidence interval; CrAg+, CrAg-positive specimens.

### Health sub-district cryptococcal antigen detection rate

At the sub-district level, only the Laingsburg local municipality did not report a single CrAg-positive result, with 33 specimens tested with a CD4 category of < 100 cells/µL, and 44 with CD4 ≥ 100 cells/µL – ≤ 200 cells/µL (refer to Online Appendix 1). All the findings reported are for the remaining sub-districts.

**FIGURE 1:**
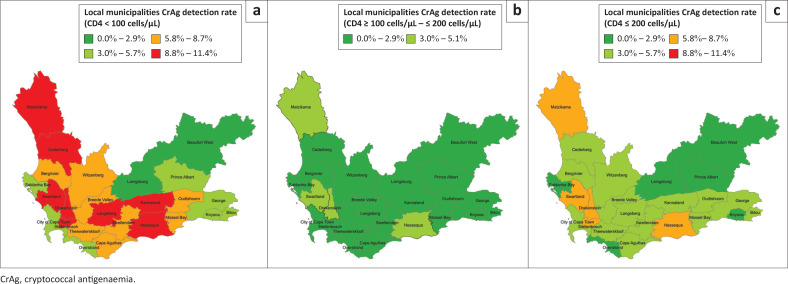
Reflexed cryptococcal antigenaemia (CrAg) detection rate by sub-district for a CD4 count (a) < 100 cells/µL, (b) ≥ 100 cells/µL – ≤ 200 cells/µL, and (c) ≤ 200 cells/µL in the Western Cape between September 2022 and May 2024.

For a count < 100 cells/µL, the CrAg detection rate ranged from 2.2% (Beaufort West) to 11.4% (Swartland). A CrAg detection rate ≥ 8.8% was reported for eight sub-districts for a CD4 count of < 100 cells/µL (Figure 1a). By comparison, for a count of ≥ 100 cells/µL – ≤ 200 cells/µL, the Prince Albert local municipality also reported no CrAg-positive results and was excluded, with findings reported for the remaining sub-districts. The CrAg detection rate ranged from 0.5% (Beaufort West) to 5.1% (Swartland). There are only four sub-districts where the CrAg detection rate for a count of ≥ 100 cells/µL – ≤ 200 cells/µL was 3.0% or higher (Figure 1b). For a count ≤ 200 cells/µL, except for Laingsberg, the sub-district CrAg detection rate ranged from 1.3% (Beaufort West) to 8.3% (Swartland). Only six sub-districts reported a CrAg detection rate in the 0.0% – 2.9% bucket (Beaufort West, Knysna, Laingsberg, Overstrand, Prince Albert, Saldanha Bay) (Figure 1c). A CrAg detection rate between 5.8% and 8.7% was reported for five sub-districts (Drakenstein, Hessequa, Matzikama, Stellenbosch and Swartland).

## Discussion

We found that the CrAg detection rate was 4.0% for extending reflexed screening to a CD4 threshold of 200 cells/µL in the Western Cape province, which is above the cost-effectiveness prevalence threshold of 3% reported by Meya et al.^19^ This suggests that the recommendations by the SAHCS and WHO are justified in this setting, given the reported CrAg detection rates.^9,13^ However, most CrAg-positive specimens were still identified with a CD4 count < 100 cells/µL. Overall, an increase in the CD4 count to a threshold of 200 cells/µL was accompanied by a decreasing CrAg detection rate.

A local study reported that the cost to find one CrAg case ranged from $589.74 to $73.72 for a detection rate of 1% to 8%.^22^ These findings suggest that the cost to find one CrAg-positive patient at a detection rate of 4.0% in the Western Cape would be $147.43 at a CD4 threshold of 200 cells/µL compared to $98.29 for a count < 100 cells/µL.^22^ This reveals that extending reflexed CrAg screening to a CD4 < 200 cells/µL incurred an incremental cost of $49.14 to find one CrAg-positive patient compared to the standard of care. Extending reflexed CrAg screening to a CD4 threshold of 200 cells/µL at the national level would double specimen volumes and costs as an additional 10.8% of total CD4 specimens have a count < 200 cells/µL, versus 9.8% < 100 cells/µL. Additional funding would have to be requested to increase the budget for laboratory expenditure, which is estimated to be $1 772 890 for 2019.^22^

Furthermore, when the Western Cape data were analysed by district, only the Central Karoo reported a CrAg detection rate below 3%. It is also, therefore, not surprising that very few CrAg-positive specimens were identified in the sub-districts within Central Karoo, namely Laingsburg, Prince Albert, and Beaufort West. In contrast, much higher CrAg detection rates were reported for the Cape Winelands and West Coast sub-districts. This emphasises that decisions to extend reflexed testing for a count of 100 cells/µL – 200 cells/µL in other settings may require a more targeted approach that is informed by prevalence data where the CrAg detection rate exceeds 3%.^19^ For the prevalence study, we could leverage remnant plasma for current CD4 testing to assess the CrAg detection rate across South Africa for a count of 100 cells/µL – 200 cells/µL. Providing these prevalence data make it possible to determine whether to extend reflexed CrAg screening based on a detection rate ≥ 3% for targeted implementation at the district level. Once identified, the LIS rules could be amended to extend reflexed CrAg screening, as was the case for the Western Cape. Such a targeted approach may result in some patients not receiving a reflexed CrAg test. However, the healthcare worker can still request a non-reflexed CrAg test for patients presenting with symptoms of CM.^4,9,13^ Using this mechanism, any patients who may be missed through the reflexed option could still be diagnosed using provider-initiated testing.

It would be imperative in areas such as the Western Cape, where the health data centre has been created to link laboratory and clinical data, to answer some important questions such as the number of CrAg-positive cases that are ART naïve.^25^ It has been locally reported that for patients in the Western Cape with CD4 counts < 50 cells/µL in 2016, 51.8% were ART experienced.^7^ It is worrying to note that these patients had previously started ART and are re-presenting with AHD.^7^ Furthermore, CrAg cases can be investigated to determine the proportion that received a lumbar puncture and went on to receive antifungal treatment.

### Limitations

A limitation of this study is that it only reported specimen-level data for reflexed CrAg testing using the laboratory data repository. Furthermore, data this research is not part of a thesis only reported for reflexed CrAg testing, which is in line with local guidelines.^26^

## Conclusion

The study findings of a CrAg detection rate of 4.0% in the Western Cape province justifies the decision to extend reflexed screening to a CD4 threshold of 200 cells/µL. However, most CrAg-positive specimens were still identified with a CD4 count < 100 cells/µL. Overall, an increase in the CD4 count up to a threshold of 200 cells/µL was accompanied by a decreasing CrAg detection rate. This emphasises that decisions to extend reflexed testing for a count of 100 cells/µL – 200 cells/µL in other settings may require a more targeted approach that is informed by prevalence data where the CrAg detection rate exceeds 3%. Furthermore, intensified approaches are required to find PLHIV with AHD and investigate the potential barriers to healthcare seeking or presentation.
